# Life cycle impacts of ethanol production from spruce wood chips under high-gravity conditions

**DOI:** 10.1186/s13068-016-0468-3

**Published:** 2016-03-05

**Authors:** Matty Janssen, Charilaos Xiros, Anne-Marie Tillman

**Affiliations:** Department of Energy and Environment, Division of Environmental Systems Analysis, Chalmers University of Technology, Rännvägen 6B, SE-41296 Göteborg, Sweden; Department of Biology and Biological Engineering, Industrial Biotechnology, Chalmers University of Technology, Kemigården 4, SE-41296 Göteborg, Sweden; School of Agricultural, Forest and Food Sciences HAFL, Bern University of Applied Sciences, Länggasse 85, 3052 Zollikofen, Switzerland

**Keywords:** Life cycle assessment, High-gravity hydrolysis and fermentation, Ethanol production, Spruce wood chips, Technology development, Global warming potential, Eutrophication potential, Acidification potential, Photochemical ozone creation potential, Renewable and non-renewable energy use

## Abstract

**Background:**

Development of more sustainable biofuel production processes is ongoing, and technology to run these processes at a high dry matter content, also called high-gravity conditions, is one option. This paper presents the results of a life cycle assessment (LCA) of such a technology currently in development for the production of bio-ethanol from spruce wood chips.

**Results:**

The cradle-to-gate LCA used lab results from a set of 30 experiments (or process configurations) in which the main process variable was the detoxification strategy applied to the pretreated feedstock material. The results of the assessment show that a process configuration, in which washing of the pretreated slurry is the detoxification strategy, leads to the lowest environmental impact of the process. Enzyme production and use are the main contributors to the environmental impact in all process configurations, and strategies to significantly reduce this contribution are enzyme recycling and on-site enzyme production. Furthermore, a strong linear correlation between the ethanol yield of a configuration and its environmental impact is demonstrated, and the selected environmental impacts show a very strong cross-correlation ($r^2>0.9$ in all cases) which may be used to reduce the number of impact categories considered from four to one (in this case, global warming potential). Lastly, a comparison with results of an LCA of ethanol production under high-gravity conditions using wheat straw shows that the environmental performance does not significantly differ when using spruce wood chips. For this comparison, it is shown that eutrophication potential also needs to be considered due to the fertilizer use in wheat cultivation.

**Conclusions:**

The LCA points out the environmental hotspots in the ethanol production process, and thus provides input to the further development of the high-gravity technology. Reducing the number of impact categories based only on cross-correlations should be done with caution. Knowledge of the analyzed system provides further input to the choice of impact categories.

**Electronic supplementary material:**

The online version of this article (doi:10.1186/s13068-016-0468-3) contains supplementary material, which is available to authorized users.

## Background

More sustainable processes for the production of biofuels continue to be the focus of ongoing technology development. The industry has been aiming at improving the energy and material efficiency of existing bio-ethanol production technologies with the goal to make these more cost competitive (for some examples see chapter 12 in [1]). This may be achieved by running the hydrolysis and fermentation processes at high concentrations of substrate, also called high-gravity conditions [2]. This may result in a reduction of water use in the process and a higher concentration of ethanol in the fermentation broth. Consequently, the energy needed during the downstream processing of the broth is reduced [3]. However, such high-gravity processes have mostly been developed using sugars derived from starch- or sucrose-containing crops such as grains, corn, sugar cane, or sugar beet [4]. Nevertheless, due to concerns about the possible competition with food production [5, 6], the development of a hydrolysis and fermentation process using a second generation feedstock, that is, a ligno-cellulosic feedstock, has been considered. Koppram et al. [2] reviewed the challenges and perspectives regarding ligno-cellulosic ethanol production at high-gravity conditions. The main challenges that need to be dealt with are (a) high concentrations of inhibitory substances that are generated during the pretreatment of the feedstock, (b) high concentrations of sugars and ethanol which in themselves are inhibitory, and (c) the high viscosity of the pretreated material in the hydrolysis and fermentation step which results in mixing and mass transfer limitations. These challenges have recently been investigated for the production of ethanol from spruce wood chips [7, 8] and the results of this research form the basis of the work presented in this paper.

Life cycle assessment (LCA) is a tool for assessing the environmental aspects and potential impacts associated with a product throughout its life cycle [9, 10]. LCA has been applied extensively to assess second generation bio-ethanol production from different kinds of feedstocks. Wood is also considered as a feedstock, and assessments have been done of ethanol production from wood via biochemical conversion [11–14]. However, to the best of our knowledge no LCA has yet been done for the production of bio-ethanol from wood using a high-gravity process technology. An LCA of bio-ethanol production based on wheat straw using a similar technology has already been done by authors of this paper [15]. This study was based on experimental work done by Cannella et al. [16, 17], and highlighted the relatively high contribution of enzyme production and use to the environmental impact of the technology under study. On-site enzyme production may be a remedy to lower this contribution as was pointed out by MacLean and Spatari [18]. Another option may be to adjust the process such that the enzyme can be recycled [19].

LCAs of second generation ethanol production have generally been done using data that describe industrial-scale operations (for instance from the well-known NREL studies [20, 21]) and thus assess the bio-ethanol life cycle at a mature development stage. LCA can however also be used to assess the environmental impacts of a process technology that is in development such as is the case in the current study. Shibasaki et al. [22] developed a method to assess technologies that are at an early stage of development using LCA, and pointed out that scale-up effects cannot be neglected when such a technology is compared to a technology that already runs at an industrial scale. Besides scale issues, Hillman and Sandén [23] identified changes in the background system as an issue that is given little attention. The background system may change not only over time due to changes in e.g., a country’s energy mix, but may also change due to the scale at which a new technology is applied, e.g., increased land use due to increased biofuel production.

Cherubini and Strømman [24] pointed out in their review of LCA studies of bioenergy systems that, on the one hand, most of these studies included global warming potential (GWP) and energy use in the impact assessment. On the other hand, a minority of the reviewed studies included impact categories like acidification and eutrophication. In the case of assessments of technologies that are at an early development stage, the inclusion of many impact categories may be questioned because of considerable uncertainty about how the technology will perform at a large scale. Instead, a possible route to avoid the assessment of many impact categories is to streamline the LCA by making use of correlations that exist between the impact categories [25, 26]. Only few studies considered land use and land use change in their impact assessment due to the lack of a widely accepted impact assessment methodology [24]. Nevertheless, impacts due to land use and land use change are considered in policies internationally. It has been argued that the carbon in biomass with a slow growth rate, e.g., trees, and with a fast growth rate, e.g., wheat straw, needs to be treated differently [27–29]. The growth rate determines how fast CO$_2$, emitted after burning the biomass for instance, is assimilated into the growing biomass. The impact of such biogenic CO$_2$ emissions is another subject of current research (see e.g., [30–32]), but a general method for assessing these impacts which are highly case specific, is difficult to develop and deploy. A widely accepted approach is therefore still lacking.

The purpose of the current LCA was to assess the environmental impact of high-gravity conditions during the hydrolysis and fermentation steps in an ethanol production process with spruce wood chips as the feedstock, at a very early development stage. Furthermore, the main factors that determine the environmental impact of the technology in development were determined. From a methodological point-of-view, the possibility of streamlining the LCA was explored. Finally, the ethanol production from spruce wood chips using high-gravity technology was compared with its production from wheat straw using a similar technology. The results of the LCA are intended to help guide the development of the technology for high-gravity hydrolysis and fermentation by providing technology developers, researchers and industry decision makers the environmental hotspots from an environmental life cycle point-of-view.

## Methods

### Description of the system under study

**Fig. 1:**
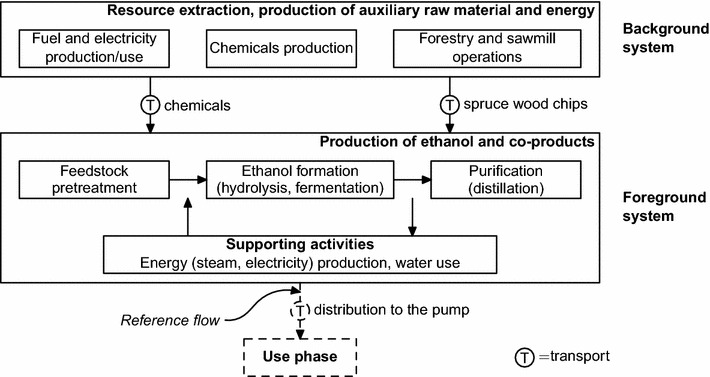
System boundary of the life cycle assessment. The *dashed lines* indicate the processes and flows that are outside the scope of this LCA

**Fig. 2:**
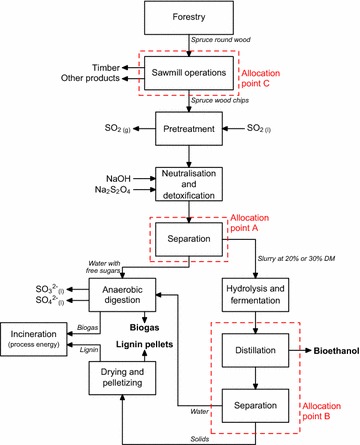
Flow diagram of the ethanol production process under study. Three allocation points are indicated in the figure

The LCA conducted in this study was defined as a cradle-to-gate system, from the extraction of spruce wood until the gate of the ethanol production plant, for a set of process configurations (see “LCA of process configurations” section) (Fig. 1). The ethanol plant was assumed to be located in Örnsköldsvik, Sweden. In order to simplify wood transport modeling, it was assumed that enough spruce wood chips are available from saw mills nearby the plant, at an average transportation distance of 25 km. Also included in the upstream activities were the production of enzymes, detoxification agents, sodium hydroxide (needed to adjust the pH to 5.0), sulfur dioxide (used in the acid-catalyzed pretreatment), and all fuel and electricity needed in the system. The model for the ethanol production process was based on the SEKAB E-Tech process [33] (Fig. 2). In this continuous process, the spruce wood chips that arrive from sawmills in the vicinity of the plant are first screened (not part of the model, see Fig. 2) and then pretreated with steam under acid-catalyzed conditions. The pretreated material is neutralized and detoxified, and water with dissolved free sugars is discharged to an anaerobic digester (AD) where biogas (consisting of 60 % CH$_4$ and 40 % CO$_2$ on a mass basis [34]) is produced as a by-product of the overall process. The pretreated material, now consisting mainly of cellulose, lignin and free C$_6$ sugars, is further processed in the enzymatic hydrolysis and fermentation steps in order to produce ethanol. The ethanol is separated and purified from the broth via distillation and molecular sieves up to 99.5 % (*v/v*). The solids in the bottom product are dried and made into pellets that mainly consist of lignin, and the water with dissolved fermentation by-products is fed to the anaerobic digester. The methane produced in the AD and the lignin pellets were assumed to be incinerated to provide the process with the required energy. A surplus of either by-product was exported. It should be noted that the non-digested cellulose was treated as a material loss (it is neither recycled nor incinerated). This was done in order to model the production of lignin pellets by-product with a high purity. The distribution of the produced ethanol to the pump and the use phase were excluded from the assessment because the focus is on the development of the new high-gravity technology for ethanol production.

### LCA of process configurations

The LCA of various high-gravity process configurations was based on the results of two sets of laboratory-scale experiments [7, 8]. The main goal of these experiments was to counteract the negative effects of inhibitory compounds that occur during the high-gravity fermentation of acid steam-pretreated spruce slurry. The two sets of experiments consisted of:

1. Twenty-one batch experiments in which four different detoxification strategies were tested using two process strategies, namely, separate hydrolysis and fermentation (SHF) and pre-saccharification and fermentation (PSSF) [7]. The four strategies that were tested were (1) adaptation of the yeast cells to the environment of the spruce slurry, (2) adaptation of the yeast cells and the use of additional nutrients, (3) detoxification of the slurry using sodium dithionite (Na$_2$S$_2$O$_4$), and (4) washing the slurry, and subsequent use of the solid fraction in fermentation. The experiments were run at 30, 34, 37, and 40 $^\circ$C.
2. Five experiments in which batch and fed-batch strategies were tested for process configurations using the simultaneous saccharification and fermentation (SSF) process strategy [8]. Two batch experiments were run to determine the fermentability of the pretreated spruce slurry and the solid fraction of this slurry. Three fed-batch experiments were run to determine the influence of feeding the solid fraction of the pre-treated slurry (the substrate), the enzyme preparation and the yeast cells. In all three experiments, the substrate was fed to the reactor, and (1) only the enzyme preparation was fed, (2) only the yeast cells were fed, and (3) both the yeast cells and the enzyme preparation were fed to the reactor. These experiments were all run at 35 $^\circ$C.

All batch experiments were run with a dry matter (DM) content of 30 % DM (corresponding to 20 % water-insoluble solids (WIS) content), except for the PSSF batch experiments in which the slurry was washed. In those experiments, the DM content was equal to the WIS content (20 %) because all water-soluble solids were removed. Furthermore, the enzyme preparation that was used in these experiments was Cellic CTec 2 and the enzyme load was 7.5 FPU g$_\mathrm{DM}^{-1}$ (9.5 mg$_\mathrm{protein}$ g$_\mathrm{DM}^{-1}$). The concentration of the yeast cells was 5 g L$^{-1}$. During the fed-batch experiments (a) the WIS content increased from 10 to 20 % WIS, (b) the yeast cell concentration decreased from 7 to 5 g L$^{-1}$, and (c) the enzyme loading decreased from 22.5 to 7.5 FPU g$_\mathrm{WIS}^{-1}$. For more detailed information about these experiments, the reader is referred to [7] and [8]. Additionally, a base case process configuration was defined for comparison purposes and a lab experiment was done for it. In this configuration, the PSSF process strategy was run at 30 °C and at a more conventional DM content of 12 % DM (equivalent to 8 % WIS), using Na$_2$S$_2$O$_4$ to detoxify the pretreated spruce slurry. This detoxification strategy was chosen because it is likely the one that would be implemented in an industrial-scale ethanol production process. Thus, in total 27 different process configurations were analyzed.

The functional unit in this assessment was 1 L of ethanol produced from spruce by a process system that uses the high-gravity hydrolysis and fermentation technology. The LCA was carried out using an attributional approach, because the purpose was to identify improvement possibilities in the technologies in development (and to compare them to the base case process configuration), and thus on what to focus in the development.

The life cycle impact assessment (LCIA) was carried out using the CML characterization method [35]. The following impact categories were used for the evaluation of the process configurations:

- GWP The main goal of the use of biofuels is to reduce the use of fossil-based fuels and thus reducing their potential impact on global warming. In this assessment, biogenic greenhouse gas (GHG) emissions were assumed to be climate neutral, and thus only the environmental impact of fossil GHG emissions of the analyzed system were taken into account.
- Eutrophication potential (EP) The use of nutrients during the production of the enzyme preparation can lead to increased eutrophication due to the emission of phosphorus and nitrogen.
- Acidification potential (AP) Combustion of fossil fuels, biogas, and lignin pellets can lead to increased acidification due to the emission of $\mathrm{SO}_2$, $\mathrm{NH}_3$ and $\mathrm{NO}_\mathrm{x}$.
- Photochemical ozone creation potential (POCP) Combustion of fossil fuels, biogas, and lignin pellets can lead to increased photochemical ozone creation due to the emission of volatile organic compounds (VOCs), CO and $\mathrm{NO}_\mathrm{x}$.

Our previous LCA study on the production of ethanol from wheat straw demonstrated that the ethanol yield is the main determinant of the environmental impact of a process configuration [15]. This was further investigated in the current study using regression analysis, and cross-correlations between the impact categories were determined. Furthermore, the environmental impact results of the current study were compared with those of the wheat straw ethanol LCA study [15] in order to determine if there is a significant difference between the use of these two feedstocks, from an environmental point of view.

A partitioning of the environmental burden based on the economic value of the main product (ethanol) and the by-products (biogas, lignin) was used (see Table 1) [36]. This was done according to the flow diagram of the SEKAB process and includes two allocation points: one (allocation point A in Fig. 2) at the neutralization and detoxification stage where water with free sugars is discharged to the anaerobic digester and all other material continues to the hydrolysis and fermentation, the pH is adjusted and the slurry is detoxified, and one (allocation point B in Fig. 2) where the ethanol and lignin pellets are separated from the remaining liquid. In the case of allocation point A, it was assumed that the prices of the fiber flow out of the neutralization and detoxification stage, and of the water stream both were 70 € t$^{-1}$ (reflecting the resource use to convert the wood spruce chips into these two streams). Furthermore, sawmill chips are a by-product of timber production with an economic value, which requires a partitioning of the environmental burden. Therefore, a third allocation point is at the sawmill operations (allocation point C in Fig. 2). The data describing these operations and the applied allocation were taken from Liptow et al. [37]: 9.6 % of the impact of electricity use in the sawmill was allocated to the sawmill chips; there was no allocation of the impact of using heat to the sawmill chips because they are not dried. Sensitivity analyses were carried out to investigate changes in the environmental impact allocated to ethanol due to varying product prices.

**Table 1:** Energy content and price of the feedstock and products of the process

	Energy content [MJ kg$^{-1}$]	Price [€ t$^{-1}$]
Spruce	19.2	60$^\mathrm{a}$
Ethanol	29.7	600$^\mathrm{b}$
Lignin	24	300$^\mathrm{c}$
Biogas (methane)	50	570$^\mathrm{d}$

Spruce is the feedstock, ethanol is the main product, and biogas and lignin pellets are the by-products

^a^Price of delivered spruce wood chips is assumed to be 60 € t$^{-1}$ (based on data from Table 3 (p. 1076) in Liptow et al. [37])

^b^ http://www.nasdaq.com/markets/ethanol.aspx

^c^Assumed to be sold as an alternative renewable fuel [57]

^d^Price is based on natural gas price for medium size industries in Sweden in 2014 [58]

## Mass and energy balances and other sources of data

The calculations of the mass and energy balances for process configurations were similar to those described by Janssen et al. [15]. The results of these calculations can be found in the Additional file 1. The final ethanol yields were the main experimental results, and were used to calculate the amount of ethanol produced from the total fermentable sugars available. These yields, expressed as a percentage of the maximum theoretical ethanol yield, were determined at the end of the fermentation steps for the various process configurations [7, 8]. It was assumed that these experimental lab data also apply to the industrial scale, i.e., the same yields and usage of enzyme and chemicals are assumed to apply. It should be noted that the total process time (hydrolysis time $+$ fermentation time) in the experiments described by Xiros and Olsson [7] varied to achieve the maximum ethanol yield. This was explicitly accounted for by calculating the energy needed for mixing the pretreated slurry during hydrolysis and fermentation. This calculation was based on lab-scale experimental data from Palmqvist and Lidén [38].

Several assumptions were made to calculate the mass and energy flows. With regard to the sawmill chips used in the process (output from forestry and sawmill operations, Fig. 1), it was assumed that (a) the sawmill chips consisted of 47 % m/m cellulose, 25 % m/m hemicellulose and 28 % m/m lignin [39], (b) there were no material losses from tree harvesting until the pretreatment of the sawmill chips, and (c) the water content of the harvested spruce was 50 %. With regard to the high-gravity process in development itself (the foreground system in Fig. 1): (a) the acid-catalyzed pretreatment was done at $p\,=\,20\,\mathrm{bar}$ and $T=212\,^\circ$C; (b) data on emissions of SO$_{2\mathrm{(g)}}$ from the process were taken from Twumasi [40]; (c) the produced biogas consisted of 60 % $\mathrm{CH}_4$ and 40 % ${\mathrm{CO}_2}$; (d) 0.35 m${_{\mathrm{CH}_4}^{3}}$ kg$_\mathrm{COD}^{-1}$ (COD = Chemical Oxygen Demand) was produced in the anaerobic digestion (AD) [41], and 85 % of the COD was converted [34]. The COD of $\mathrm{C}_5$ sugars is 1.052  g$_\mathrm{COD}$ g${_{\mathrm{C}_5}^{-1}}$ and of $\mathrm{C}_6$ sugars 1.067 g$_\mathrm{COD}$ g${_{\mathrm{C}_6}^{-1}}$; (e) the sulfur contained in $\mathrm{SO}_2$ used during pretreatment and Na$_2$S$_2$O$_4$ used for detoxification of the pretreated slurry left the system in the form of sulfite and sulfate via the anaerobic digester (Fig. 2) and it was assumed that this resulted in no further impacts; (f) the power consumption during hydrolysis and fermentation of the pretreated slurry was 1.5 kW kg${_\mathrm{WIS}^{-1}}$ [38]; (g) the energy input for distillation was taken from Galbe et al. [42] (see p. 319, Fig. 6), which includes preheating of the feed to 80 $^\circ$C; (h) the solids after distillation (Fig. 2) were assumed to be dried by pressing up to a dry matter content of 50 %. These solids were then further dried up to 90 % DM; and (1) it was assumed that methane is burned first with a 90 % efficiency that lignin is burned next with a 75 % efficiency, and that additional fossil fuel mix, if needed in the process configuration, is burned with a 90 % efficiency. Using the methane for process energy purposes was modeled using the ecoinvent process ‘heat, at cogen with biogas engine, allocation exergy’ [43]. The combustion of the lignin pellets was modeled using the ‘Combustion, dry wood residue, AP-42’ process from the US LCI database [44]. This process was adjusted in order to account for the heating value of lignin (assumed to be 24 MJ kg${_\mathrm{lignin}^{-1}}$) and the combustion efficiency of lignin. The additional fossil fuel mix was assumed to be the Swedish energy fossil fuel mix [45].

Several choices also had to be made for modeling forestry and sawmill operations, and enzymes and chemicals production (the background system in Fig. 1):

1. The production of $\mathrm{SO}_2$ was modeled using the ecoinvent process ‘sulfur dioxide production, liquid’ [46]. 18 g${_{\mathrm{SO}_2}}$ kg${_\mathrm{DM}^{-1}}$ of spruce wood chips was added during the pretreatment.
2. Production of Na$_2$S$_2$O$_4$ was modeled using the ecoinvent process ‘sodium dithionite, anhydrous, at plant’ [46]. 6 g${_{\mathrm{Na}_{2}\mathrm{S}_{2}\mathrm{O}_{4}}}$ kg${_\mathrm{DM}^{-1}}$ of pretreated spruce was added in the experiments that used Na$_2$S$_2$O$_4$ in order to reduce the effect of inhibitors on the fermentation. In the base case experiment, 14.5 g${_{\mathrm{Na}_{2}\mathrm{S}_{2}\mathrm{O}_{4}}}$ kg${_\mathrm{DM}^{-1}}$ of pretreated spruce was added.
3. The production of NaOH was modeled using the ecoinvent process ‘sodium hydroxide, 50 % in H$_2$O, production mix, at plant.’ The environmental impact of this process is allocated according to the masses of the different products (52.3 % NaOH, 46.4 % ${\mathrm{Cl}_2}$ and 1.3 % ${\mathrm{H}_2}$). Mass allocation was applied in this process because the amounts produced of the three chemicals can be clearly determined [46]. 7 g${_\mathrm{NaOH}}$ kg${_\mathrm{DM}^{-1}}$ of pretreated spruce was added in the experiments in order to adjust the pH to a level that is favorable to the hydrolysis and fermentation of the pretreated spruce.
4. The production of the Cellic CTec2 enzyme preparation was modeled using the life cycle inventory data published by Liptow et al. (see Table 5, p. 1077) [37] and took place in Kalundborg, Denmark. The enzyme loads used in the different experiments are given in “LCA of process configurations” section.
5. Additional nutrients (see “LCA of process configurations” section) were assumed to be yeast extract, and their production was modeled using the ecoinvent process ‘yeast paste, from whey, at fermentation’ [43]. 17 g$_\mathrm{yeast\,extract}$ kg${_\mathrm{DM}^{-1}}$ was added in the experiments that made use of these additional nutrients.
6. The fossil fuel mix used as additional fuel for the ethanol production process was assumed to be the Swedish mix from 2011 and consists of 11 % coal, 82 % oil and 7 % natural gas [45]. This fuel was used for steam production.
7. The electricity used by the ethanol production process was produced in Sweden, and was modeled with the ecoinvent process ‘electricity, production mix SE’ [47].

The LCA software openLCA version 1.3 [48] was used to model the complete ethanol production system according to Fig. 1 (both the foreground and background systems) for the different process configurations, and to calculate their environmental impacts. The mass and energy balance results for the process configurations and the models used for describing the background system were thus integrated in this software.

## Results and discussion

### Impacts of the process configurations

**Fig. 3:**
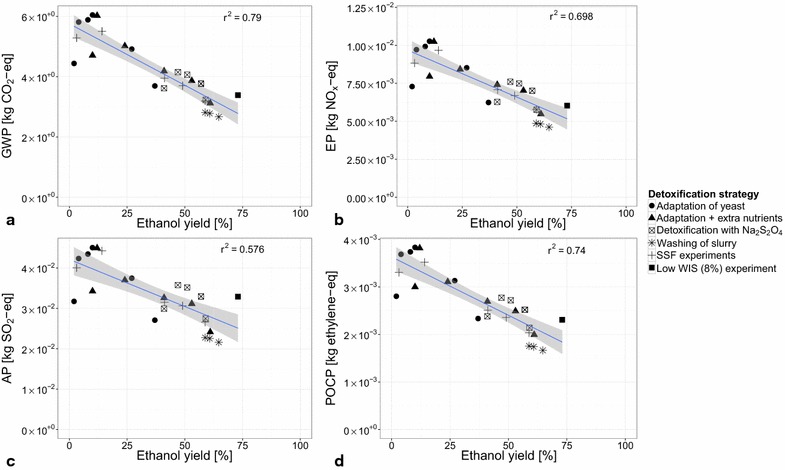
Correlation between the ethanol yield of the process configurations and their environmental impacts. The impact categories are: **a** global warming potential (GWP); **b** eutrophication potential (EP); **c** acidification potential (AP); **d** photochemical ozone creation potential (POCP). The linear trend line (in *blue*) and the 95 % confidence intervals (in *gray*) are given. The correlation coefficients ($r^2$) are mentioned in each* graph*

**Fig. 4:**
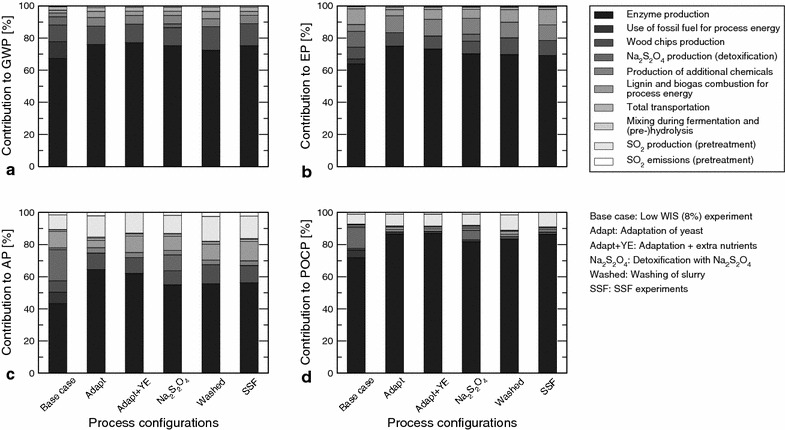
Contribution analysis of the tested detoxification strategies. The impact categories are: **a** global warming potential (GWP); **b** eutrophication potential (EP); **c** acidification potential (AP); **d** photochemical ozone creation potential (POCP). The contributions of the different processes are given for the best performing process configuration of the different detoxification strategies (the other configurations show similar results) and the base case process configuration

All process configurations described in “LCA of process configurations” section were analyzed and the main results are summarized in Figs. 3 and 4. Figure 3 shows the relationship between the four environmental impact categories that were analyzed and the ethanol yield of the tested process configurations. Figure 4 shows the contributions of the different foreground and background processes involved in the bio-ethanol production to the environmental impacts of the different detoxification strategies.

Visual inspection of the graphs in Fig. 3 followed by a linear regression analysis revealed that there is a strong correlation between the ethanol yield of a process configuration and its environmental impacts: the higher the ethanol yield, the lower the environmental impacts. This result may be expected because a higher ethanol yield implies a lower use of both the renewable and non-renewable resources needed to produce ethanol. The environmental impact categories show a weak overall correlation with the temperature at which the process configuration is run (30, 34, 37, and 40 $^\circ$C, see “LCA of process configurations” section) (see the results in the Additional file 1). Scale-up effects were not taken into account in the analysis of the process configurations (see “Mass and energy balances and other sources of data” section). It is likely that the environmental impacts will change when these are taken into account. However, the correlations between the environmental impacts and ethanol yield, as depicted in Fig. 3, are expected to persist.

Enzyme production and use is the dominant contributor to all environmental impact categories for all of the process configurations that were tested (Fig. 4). This is due to the fossil energy needed during the production of the enzyme [37]. The explicit inclusion of the electrical power input for mixing of the slurry during the hydrolysis and fermentation does not lead to a significant change in the environmental impact of the process. The emission of ${\mathrm{SO}_2}$ during the pretreatment step also does not result in a significant impact because this emission is small (0.14 g kg${_\mathrm{DM}^{-1}}$). However, the production of ${\mathrm{SO}_2}$ that is used during the pretreatment process contributes to the acidification (AP) and POCPs (approx. 13 and 8 % on average, respectively). All the sulfur that is added to the process ends up in the AD in the form of sulfite and sulfate (Fig. 2). The following two subsections discuss the specific results for the process configurations using the SHF and PSSF process strategies, and the process configurations using the SSF process strategy, respectively (see the description of experiments in “LCA of process configurations” section).

#### SHF and PSSF process configurations

The process configurations [7] with the lowest environmental impact are those that use washing of the slurry as the detoxification strategy (Fig. 3). This may be explained by the lower DM content at which these configurations are run (20 % DM instead of 30 % DM) and by the removal of inhibitors, both resulting in a higher ethanol yield [17]. Furthermore, significant amounts of biogas are produced in these configurations because the free sugars that are generated during the pretreatment are now fed to the anaerobic digestion (Fig. 2). The produced biogas is subsequently incinerated to provide the process with energy, which results in less incineration of lignin. A larger amount of lignin pellets thus is produced as a by-product which consequently carries a larger share of the environmental burden of the process.

The production and use of ${\mathrm{Na}_2\mathrm{S}_2\mathrm{O}_4}$ contributes 13 % to AP and 7 % to POCP on average, not including the base case configuration (called ‘Low WIS (8 %) experiment’ in Fig. 3). The increased use of ${\mathrm{Na}_2\mathrm{S}_2\mathrm{O}_4}$ by the base case results in a contribution of 19 % to AP and 13 % to POCP (Fig. 4). The contribution to AP is caused by ${\mathrm{SO}_2}$ emissions, and to POCP by emissions of volatile organic compounds (VOCs). Adding ${\mathrm{Na}_2\mathrm{S}_2\mathrm{O}_4}$ to the fermentation leads to similar yields but less biogas is produced in these configurations. Consequently, a larger part of the environmental burden is allocated to the ethanol product because more lignin needs to be incinerated to provide the process with sufficient energy. This points out not only that the yield of ethanol production should be improved, but also that the production of biogas is of importance, from an environmental point of view.

In the case of the process configurations that were run at 30 % DM, for each detoxification strategy (adaptation of yeast, adaptation of yeast and use of additional nutrients, detoxification with ${\mathrm{Na}_2\mathrm{S}_2\mathrm{O}_4}$) the configuration running with the SHF process strategy at 30 $^\circ$C has the lowest environmental impact. Besides an apparent temperature effect, SHF using commercial enzyme preparations with high $\beta$-glucosidase content has been recently proven very efficient, since both fermentation and hydrolysis can then be performed under optimized conditions [7]. It should be noted that, although the base case configuration [‘Low WIS (8 %) experiment’ in Fig. 3] has the highest yield, it has a higher environmental impact than might be expected. This is due to its use of additional fossil fuel because not enough biogas and lignin are produced to meet its energy needs. It is the only configuration that is in need of this additional fuel use. This suggests that at high ethanol yields the concentration of ethanol in the fermentation broth needs to be sufficiently high in order to avoid additional fuel use. Furthermore, the base case configuration uses significantly more ${\mathrm{Na}_2\mathrm{S}_2\mathrm{O}_4}$ than the other configurations that use addition of this chemical to the fermentation as a detoxification strategy (14.5 vs. 6 g${_{\mathrm{Na}_2\mathrm{S}_2\mathrm{O}_4}}$ kg${_\mathrm{DM}^{-1}}$). This results, in particular, in a higher AP (Fig. 3c) than expected.

The process configuration with the lowest ethanol yield (adaptation of yeast, SHF at 40 $^\circ$C) has a lower environmental impact than expected. In this case, the ethanol yield is so low that all of the process energy is supplied by the methane that is generated by the process, and that all of the lignin thus leaves the system as a by-product of the process. Moreover, the amount of non-converted free sugars that go to the AD is so high that there is an excess of methane produced. Therefore, there also is methane by-product to which a part of the environmental impact is allocated leading to a lower environmental impact allocated to ethanol than expected.

#### SSF process configurations

The environmental impacts of the fed-batch SSF process configurations [8] are similar to those of the batch SHF and PSSF process configurations that have similar ethanol yields (Fig. 3). This result suggests that using a fed-batch process strategy which avoids the use of additional chemicals (${\mathrm{Na}_2\mathrm{S}_2\mathrm{O}_4}$, nutrients) to deal with the toxicity of the pretreated material is feasible from an environmental point of view. Furthermore, the batch SSF process configurations have a similar impact as the SHF and PSSF batch configurations at similarly low yields (Fig. 3). It should be noted that the final enzyme load in these experiments (7.5 FPU g${_\mathrm{WIS}^{-1}}$) is lower than in the case of the SHF and PSSF process configurations (7.5 FPU g${_\mathrm{DM}^{-1}}$). This does however neither lead to a lower environmental impact (Fig. 3), nor to a lower contribution of the enzyme production and use to the environmental impact (Fig. 4). A trade-off exists between the enzyme load and the final ethanol yield [15].

### Cross-correlations between impact categories

**Fig. 5:**
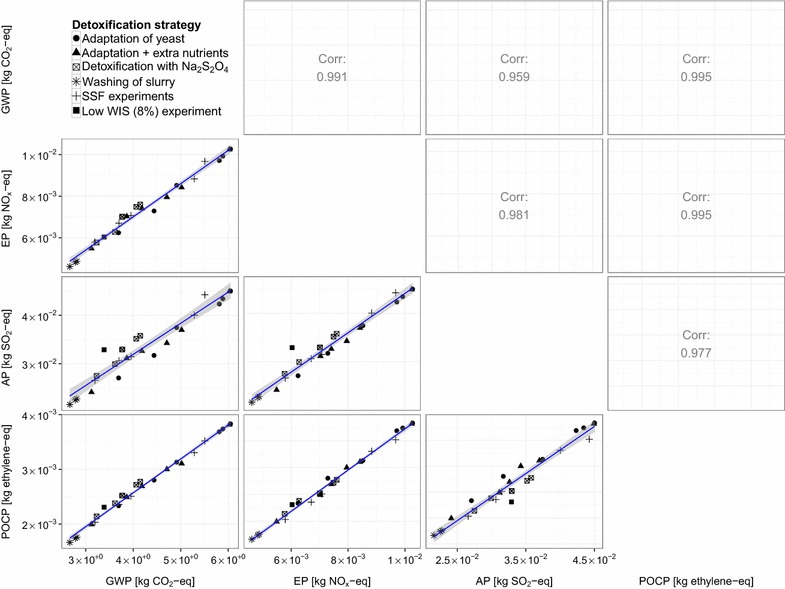
Cross-correlations between the environmental impacts of the process configurations. The *graphs* above the diagonal of the* matrix plot* show the correlation coefficients of the corresponding* graphs* below the diagonal. For instance, the correlation coefficient for AP and GWP is 0.959

There is a very high cross-correlation between the results of the four environmental impact categories that were considered in this LCA ($r^2 > 0.9$ in all cases) (Fig. 5). This suggests that using one of these impact categories is sufficient to accurately describe the environmental performance of a process configuration based on its ethanol yield. It is suggested that in this case the GWP is the impact category that can be used to predict the values of the other impact categories because it has the strongest correlation with the ethanol yield (Fig. 3).

### Sensitivity analyses

#### Mass and energy balances

The base case process configuration is the only configuration that is in need of additional fuel to meet its energy needs. It has been assumed that this additional energy is delivered in the form of fossil fuel, and amounts to 3.1 MJ L${_\mathrm{ethanol}^{-1}}$. If, instead of this fossil fuel, biomass was to be used (assuming there is enough biomass available close to where the ethanol plant is situated), then the GWP would be reduced by 7.5 %. AP and POCP would be reduced by approx. 3 and 4 %, respectively. The EP however would slightly increase (approx. 0.4 %) due to a relatively high contribution of biomass combustion to this impact category.

The consumption of fossil fuel may also be reduced by increasing the combustion efficiency of the renewable fuels generated by the process itself, that is, lignin and methane. The combustion efficiency of methane is not considered here because it is already high at 90 %. The combustion efficiency of lignin needs to increase from 75 to 87 % in order to completely avoid the use of fossil fuel. Improving the energy efficiency of the pretreatment by 26 % would also lead to the elimination of fossil fuel use. Both improvements lead to a similar reduction of the GWP (approx. 7.5 %) as in the case of replacing fossil fuel with biomass at a combustion efficiency of 75 %. These process improvements will also positively affect the GWP of all the other process configurations. For instance, in the case of the configuration with the highest yield (washing of slurry, PSSF at 40 $^\circ$C), increasing the lignin combustion efficiency to 87 % would increase the amount of lignin product from 0.72 kg L${_\mathrm{ethanol}^{-1}}$ to 0.84 kg L${_\mathrm{ethanol}^{-1}}$. This would consequently result in a reduction of the GWP allocated to the ethanol product by approx. 5 %.

The energy input for distillation was taken from Galbe et al. [42] (see p. 319, Fig. 6). This figure was however generated from experimental data which assumed 10 % WIS, while the results presented here are based on experiments run at 20 % WIS (see “LCA of process configurations” section). This will likely affect the energy demand of the distillation, and it was assumed that it increases by 10 %. In the case of the process configurations with the highest yields at 20 % DM and 30 % DM (washing of slurry, PSSF at 40 $^\circ$C and adaptation of yeast + extra nutrients, SHF at 30 $^\circ$C, respectively), this leads to an increase of the GWP allocated to the ethanol product by approx. 1.5 %. In these two cases, there is enough lignin by-product to provide the increased energy demand of the process. However, there are also process configurations that produce little lignin by-product (at a high ethanol yield) such as the configuration that uses ${\mathrm{Na}_2\mathrm{S}_2\mathrm{O}_4}$ to detoxify the pretreated spruce wood chips, and is run using the PSSF process strategy at 30 $^\circ$C (see Additional file 1: Table S1). In this case, there still is enough lignin produced to meet an increased distillation energy demand of 10 %. The distillation energy demand would have to increase by 27 % before additional fossil fuel is needed to meet the energy demand of the process.

#### Reduction of water and energy use

One of the expected advantages of using of high-gravity conditions during the hydrolysis and fermentation is the reduction of the amount of water in the process and subsequent reduction of energy use during the downstream processing of the fermentation broth. The amount of water that needs to be removed during downstream processing in the base case process configuration (8 % WIS) is approx. 25 L L${_\mathrm{ethanol}^{-1}}$. In the case of the process configuration run at 30 % DM with the highest yield (detoxification with ${\mathrm{Na}_2\mathrm{S}_2\mathrm{O}_4}$, SHF at 30 $^\circ$C), this amount of water is reduced to approx. 10 L L${_\mathrm{ethanol}^{-1}}$. This amount goes up with decreasing yield, and surpasses 25 L L${_\mathrm{ethanol}^{-1}}$ (base case) when the ethanol yield is lower than approx. 24 % (and thus a yield loss of approx. 50 % compared to the base case configuration). This means that out of the 27 tested process configurations there are nine configurations that have an increased water use compared to the base case configuration (see Additional file 1: Table S3). In the case of the process configurations that use washing of the slurry (run at 20 % DM) as the detoxification strategy, the amount of water that needs to be removed is approx. 15 L L${_\mathrm{ethanol}^{-1}}$.

**Fig. 6:**
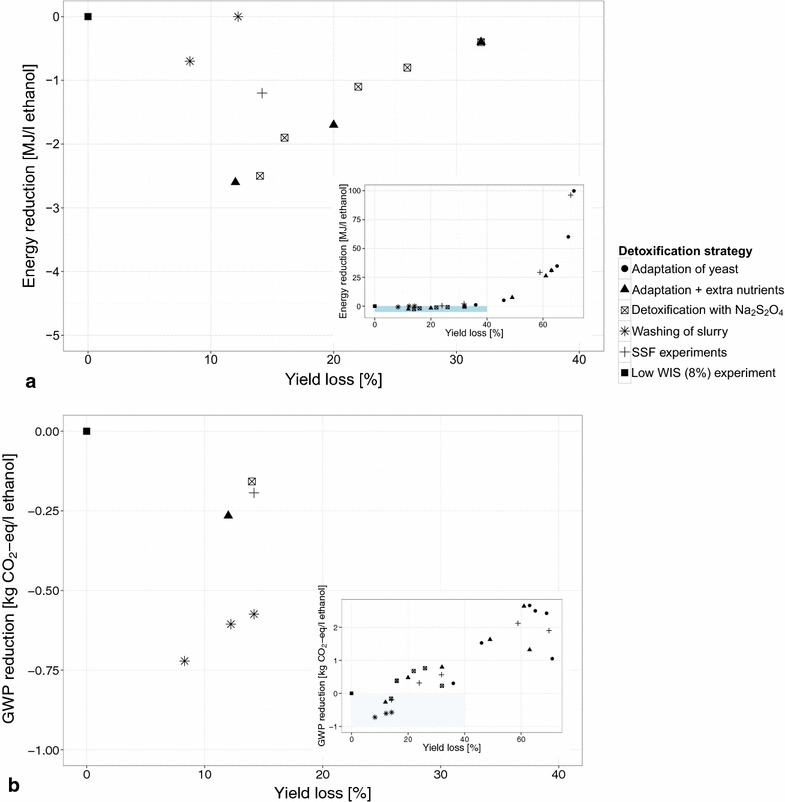
Trade-off between yield loss and reduction of energy use and GWP. **a** shows the energy reduction in the downstream processing, and **b** the reduction of the global warming potential (GWP) of the process configurations. The inset *graphs* show the results for all of the process configurations. In *blue*, the enlarged area depicted in the* main graph* is indicated

There are 12 process configurations that show a reduction in energy use during downstream processing when compared to the base case configuration (Fig. 6a). The configuration with the highest ethanol yield of those run at 30 % DM (adaptation + additional nutrients, SHF at 30 $^\circ$C) shows the greatest reduction at $-$2.6 MJ L${_\mathrm{ethanol}^{1}}$. This does however not result in the greatest GWP reduction ($-$0.27 kg ${\mathrm{CO}_2}$-eq) (Fig. 6b). The configurations using washing of the slurry as the detoxification strategy show the greatest GWP reduction ($-$0.58 kg to $-$0.72 kg ${\mathrm{CO}_2}$-eq) (see also Fig. 3a) despite their greater water use. The main reason for this is their greater methane production and consequently smaller allocation of environmental impact to ethanol (see “SHF and PSSF process configurations” section). It should be noted that only six configurations have a lower GWP than the base case configuration. Reduced energy and water use thus does not always lead to a reduction in GWP. The ethanol yield or yield loss compared to the base case appears to play a more important role in determining the GWP.

#### Allocation of impacts

The ethanol future price is volatile and this affects the environmental impact that is allocated to the ethanol product. All of the impact categories show a significant sensitivity to varying the price of ethanol. Changing this price from 600 to 415 € t${_\mathrm{ethanol}^{-1}}$ and from 600 to 825 € t${_\mathrm{ethanol}^{-1}}$ leads to a decrease of 2–30 % and an increase of 1–35 % of the impact results, respectively. The configurations with the lowest yields show the greatest sensitivity to these price changes. Only in the case of the process configuration with the lowest yield (see “SHF and PSSF process configurations” section), the price of methane affects the impact allocated to ethanol. The impact results show however a low sensitivity to changes in the methane price: an increase in the price from 570 to 750 € t${_{\mathrm{CH}_4}^{-1}}$ results in a reduction of the impacts allocated to ethanol by 1.7 %.

The water stream going from the detoxification and neutralization step to the anaerobic digester (Fig. 2) may be considered as a waste stream. As a result of this, no economic value is given to this stream and all of the environmental burden is allocated to the fiber stream leaving this step to the hydrolysis and fermentation step. The impact results are not sensitive to this and show only small increases ranging from 0.5 to 0.8 % for GWP, 0.4–0.6 % for EP, 0.9–1.4 % for AP, and 0.4–0.7 % for POCP.

### Scenario analyses

#### Use of PEI as a detoxifying agent

The use of polyethylenimine (PEI) as a detoxifying agent was tested by Cannella et al. [49]. Three process configurations were assessed and were based on the three experiments described in [49]:

1. SHF with PEI added to the slurry before hydrolysis in one case, and PEI added after hydrolysis in the other case. The hydrolysis took place at 50 $^\circ$C for 72 h and the fermentation at 34 $^\circ$C for 96 h, and
2. SSF with PEI added to the slurry before the SSF starts. The SSF was run at 34 $^\circ$C for 168 h.

The experiments were run at 19 % WIS. The enzyme preparation used was Cellic CTec2 and the enzyme load was 7.5 FPU g${_\mathrm{WIS}^{-1}}$. The concentration of the yeast cells was 6 g kg${_\mathrm{WIS}^{-1}}$. PEI (average molecular weight of 60 $\times$ 10$^{3}$ g mol$^{-1}$) was assumed to be produced via the polymerization of aziridine [50], and aziridine is formed from monoethanolamine via the Wenker process [51]. These two reaction steps were modeled in openLCA, and it was assumed that the impact of the polymerization of aziridine is negligible. The ecoinvent process ‘monoethanolamine, at plant’ was used to model the production of monoethanolamine [46]. 15 g${_\mathrm{PEI}}$ kg${_\mathrm{DM}^{-1}}$ of pretreated spruce was added in the three experiments. For a more detailed description of these experiments and their results, see [49].

**Fig. 7:**
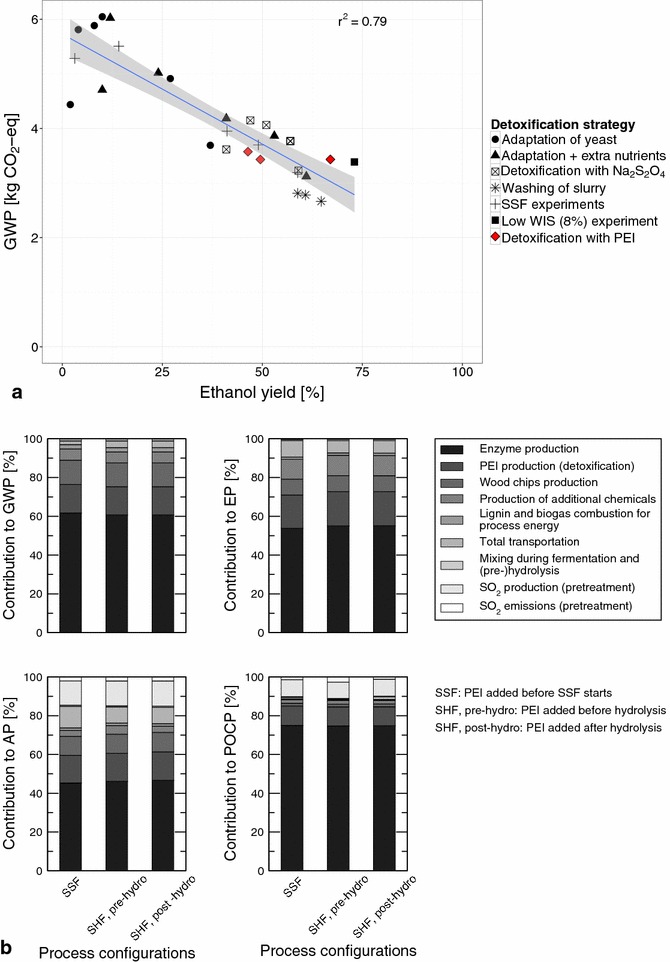
Detoxification with PEI. **a** shows the comparison with global warming potentials (GWP) of the other tested configurations, and **b** shows the contribution analysis for the tested configurations. The calculated linear trend (in *blue*) and confidence interval (in *gray*) that are shown do not include the results of the configurations that use PEI as the detoxifying agent

The results for the GWP of these three configurations (Fig. 7a, indicated with red diamonds) show that they perform similarly to the other tested configurations, and follow a similar trend. The results of the three configurations were not included in the calculation of the linear trend (this trend is the same as shown in Fig. 3a). This indicates that the resulting ethanol yield to a greater extent determines the resulting environmental impact than the detoxifying agent that is used.

The production and use of PEI as a detoxifying agent have a higher environmental impact than the production and use of ${\mathrm{Na}_2\mathrm{S}_2\mathrm{O}_4}$ for all impact categories considered. In the case of the GWP, its contribution is 6 times higher (approx. 15 vs. 2.5 %) [Figs. 4a (bar for ‘Detoxification with ${\mathrm{Na}_2\mathrm{S}_2\mathrm{O}_4}$’), 7b (bar chart for GWP)]. Cannella et al. [49] however showed that PEI can be recycled at least 5 times without a loss of efficiency. Assuming a 100 % recycling rate of PEI, this would decrease e.g., the GWP of the process configurations by approx. 12 %. Another difference with the configurations that use ${\mathrm{Na}_2\mathrm{S}_2\mathrm{O}_4}$ as the detoxifying agent is the enzyme load which is 7.5 FPU g${_\mathrm{WIS}^{-1}}$ in the case of using PEI, and 7.5 FPU g${_\mathrm{DM}^{-1}}$ in the case of using ${\mathrm{Na}_2\mathrm{S}_2\mathrm{O}_4}$ as the detoxifying agent. As a result, the contribution of enzyme production and use are approx. 61 vs. 76 %, respectively, in case of the GWP.

It should be noted that the ethanol yields for the PEI configurations as shown in Fig. 7 are lower than those reported by [49]. This is because the yield numbers shown here were imputed based on the inventory calculations (mass and energy balances) done for the LCA and the measured ethanol concentrations by [49]. The calculated amount of available sugars is different from those in the experiments because the assumed composition of the wood feedstock is different.

#### Enzyme production and use

Enzyme production and use are the largest contributors to all environmental impacts studied for all process configurations tested (67–77 % for GWP, 64–75 % for EP, 43–64 % for AP, and 72–87 % for POCP, see Fig. 4) and thus can be a target for reducing the environmental impact of ethanol production. Therefore, analyses were done of the situations in which enzyme is recycled and in which the needed enzyme is produced on-site. These analyses were done for the base case configuration and for the configurations run at 20 % DM and 30 % DM with the highest ethanol yields.

It was assumed that 25 % of the activity of the added enzyme is recycled [15] from the solids/water separation step (after distillation, see Fig. 1 in [52]) back to the hydrolysis and fermentation step (see Fig. 2), and that this does not result in a reduction of the ethanol yield. The results show that a significant improvement can be made by recycling the enzyme and thus lowering the consumption of fresh enzyme preparation (Table 2). This was also demonstrated for the case of using straw as the feedstock for ethanol production under high-gravity conditions [15].

**Table 2:** Reduction in the environmental impacts due to enzyme recycling

Process configuration	Reduction of environmental impacts			
	GWP [%]	EP [%]	AP [%]	POCP [%]
Base case	19	20	13	20
Highest yield at 20 % DM	18	18	14	21
Highest yield at 30 % DM	20	17	14	22

Impacts of an enzyme recycling rate of 25 % were compared with the impacts of no enzyme recycling. This was done for the: a. base case (detoxification with ${\mathrm{Na}_2\mathrm{S}_2\mathrm{O}_4}$, PSSF at 12 % DM and 30 °C; b. process configuration with the highest yield at 20 % DM (washing of slurry, PSSF at 40 $^\circ$C); c. process configuration with the highest yield at 30 % DM (adaptation of yeast + extra nutrients, SHF at 30 °C)

Modeling on-site enzyme production was based on data from Humbird et al. (Tables 18, 21) [21]. Furthermore, it was assumed that a fraction of the pretreated feedstock is used to produce the enzyme, and that, if needed, molasses is added as an additional sugar source. The use of this molasses was modeled with the ’molasses, from sugar beet, at sugar refinery -CH’ process from the ecoinvent database [53]. The enzyme in solution (at 50 g L$^{-1}$) is fed to the hydrolysis and fermentation step, and unused pretreated wood left after the enzyme production process (cellulose and lignin) is fed to the anaerobic digester where it is converted into biogas. It was assumed that the ethanol yield does not change when using the enzyme that is produced on-site. The results show that on-site enzyme production results in a significant decrease of the environmental impact (Table 3), and is mostly due to the elimination of fossil energy use during enzyme production. For instance, the GWP of enzyme production is reduced by approx. 80 % when moved on-site. Furthermore, extra biogas is produced from the pretreated wood that is not broken down during the enzyme production. This extra biogas is burned for the energy needs of the process and compensates for a lower production of lignin pellets, which is due to the fact that part of the lignin flows to the anaerobic digester from the enzyme production. It should be noted that the base case configuration continues to use fossil fuel to meet its energy needs in the case of on-site enzyme production. This fossil fuel use contributes approx. 27 % to the GWP of the base case configuration. If 30 % of the lignin that flows from the enzyme production to the anaerobic digester can be recovered and burned, the use of this fuel will be avoided.

**Table 3:** Reduction in the environmental impacts due to on-site enzyme production

Process configuration	Reduction of environmental impacts			
	GWP [%]	EP [%]	AP [%]	POCP [%]
Base case	59	53	32	67
Highest yield at 20 % DM	62	68	44	77
Highest yield at 30 % DM	65	69	51	85

Impacts of on-site enzyme production were compared with the impacts of off-site enzyme production. This was done for the: a. base case (detoxification with ${\mathrm{Na}_2\mathrm{S}_2\mathrm{O}_4}$, PSSF at 12 % DM and 30 $^\circ$C); b. process configuration with the highest yield at 20 % DM (washing of slurry, PSSF at 40 $^\circ$C); c. process configuration with the highest yield at 30 % DM (adaptation of yeast + extra nutrients, SHF at 30 $^\circ$C)

#### Future energy system

The changes in the process configurations analyzed in “Enzyme production and use” section are examples of changes in the foreground system. The environmental impact of the process configurations may however also change due to changes in the background system (Fig. 1). One such change is change in the energy system over time. In this case, the anticipated change in the share of fossil fuel in the energy mix in Denmark and its influence on the GWP of enzyme production (assumed to take place in Kalundborg, Denmark) was analyzed. Not only does this share change, also the fossil fuel mix itself changes (largely replacing coal with natural gas, but maintaining oil use). This anticipated change was based on data found in [54] and [55]. It is relevant to consider the background energy system since the technology under study will be implemented at a point in the future when a change in this system has been realized. The analysis was done for the base case configuration and for the configurations run at 20 % DM and 30 % DM with the highest ethanol yields. The results show that the anticipated change in the Danish energy system has a significant influence on the GWP of the ethanol production and is reduced by approx. 30 % for the analyzed configurations when the share of fossil energy is reduced from 80 to 50 % (Table 4). It should be noted that the base case configuration also uses fossil fuel (Swedish fossil energy mix) for process energy, and that this may also be replaced leading to a further decrease of the GWP. Combining this result with the result from “Enzyme production and use” section points out that by reducing enzyme use and by cleaner production of enzyme, either on- of off-site, a significantly decreased environmental impact of ethanol production under high-gravity conditions can be achieved.

**Table 4:** Global warming potentials (GWP) due to projected changes in the Danish energy mix

Process configuration	Fossil share in energy mix		
	80 %	67 %	50 %
	GWP [kg${_{\mathrm{CO}_{2^\mathrm{{-eq}}}}}$ L${_\mathrm{ethanol}^{-1}}$]		
Base case	3.4	3.0	2.4
Highest yield at 20 % DM	2.7	2.4	1.9
Highest yield at 30 % DM	3.1	2.7	2.1

This was done for the: a. base case (detoxification with ${\mathrm{Na}_2\mathrm{S}_2\mathrm{O}_4}$, PSSF at 12 % DM and 30 $^\circ$C); b. process configuration with the highest yield at 20 % DM (washing of slurry, PSSF at 40 $^\circ$C); c. process configuration with the highest yield at 30 % DM (adaptation of yeast + extra nutrients, SHF at 30 $^\circ$C)

### Comparison with wheat straw as a feedstock

Janssen et al. [15] performed an LCA of the production of ethanol from wheat straw under high-gravity conditions. As in the current study, the environmental impacts of a range of process configurations were assessed and compared. It was established in [15] that the ethanol yield of a process configuration affects both the renewable energy use (REU) (the amount of feedstock needed) and non-renewable energy use (NREU) (primarily the use of fossil energy during enzyme production) and their related emissions. REU and NREU therefore ultimately determine the environmental impact of a process configuration independent of the feedstock, and are thus good indicators to compare the use of the two feedstocks.

**Fig. 8:**
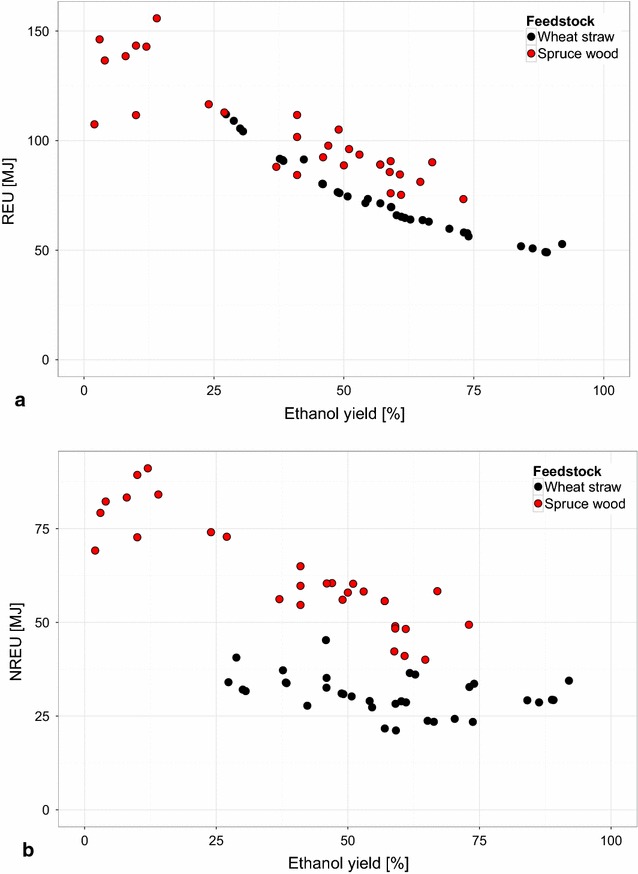
Comparison of renewable and non-renewable energy use allocated to ethanol produced from wood and straw. **a** shows the renewable energy use (REU) and **b** the non-renewable energy use (NREU) allocated to ethanol produced by a process configuration

**Fig. 9:**
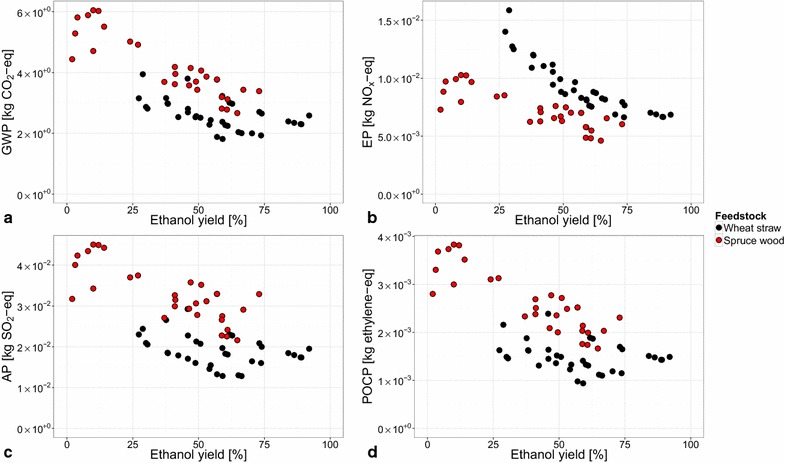
Comparison of environmental impacts using wood and straw for ethanol production under high-gravity conditions. The impact categories are: **a** global warming potential (GWP); **b** eutrophication potential (EP); **c** acidification potential (AP); **d** photochemical ozone creation potential (POCP)

In the case of both feedstocks, the REU and NREU allocated to the ethanol produced show a declining trend with increasing ethanol yield, as can be expected (Fig. 8). The allocated REU and NREU at a given ethanol yield are in general higher for the case of using wood as the feedstock. However, when considering the resulting GWP for both feedstocks, the difference between the trends for the two feedstocks appears to be less distinct for the range of ethanol yields that are overlapping (approx. 25–75 %) (Fig. 9a). The difference was statistically tested over this range of ethanol yields and it can be concluded that the two feedstocks perform similarly when considering GWP ($\alpha \ll$ 0.01).

Based on the suggestion made in “Cross-correlations between impact categories” section to use GWP values for predicting the values of the other impact categories, and based on the main contribution of enzyme production and use in the case of both feedstocks (approx. 75 % for wood, and 80 % for straw), it is expected that the feedstocks also perform similarly when considering AP and POCP. This was confirmed with a visual inspection (Fig. 9c, d, respectively). This is however not the case for EP because in the case of ethanol production from wheat straw fertilizer is used during the cultivation of wheat, which increases the EP. This leads to a lower relative contribution of the production and use of enzyme to EP [15]. Using wood as the feedstock results in a lower EP than in the case of using straw as feedstock (Fig. 9b). This result also points out that a comparison of agricultural and forestry feedstocks needs to include EP next to GWP as impact categories.

**Fig. 10:**
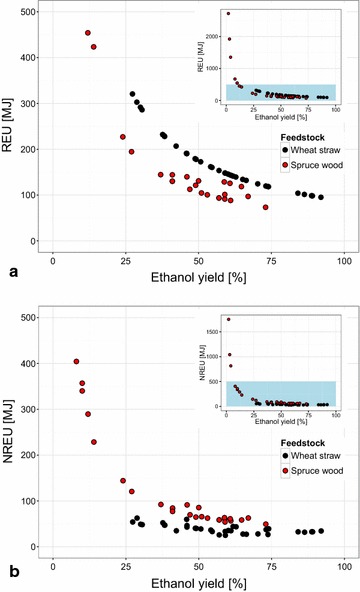
Comparison of total renewable and non-renewable energy use for production of ethanol and by-products from wood and straw. **a** shows the total renewable energy (REU), and **b** the total non-renewable energy (NREU) used by a process configuration for the production of 1 L of ethanol. The inset *graphs* show the results for all of the process configurations. In *blue*, the enlarged area depicted in the* main graph* is indicated

The total amount of REU and NREU used to produce 1 L of ethanol and its by-products (lignin and methane in the case of wood, lignin and molasses in the case of straw [15]), that is, the non-allocated values of the REU and NREU for the two feedstocks, give an indication of the total extracted energy [56] of the process under study (Fig. 2). On the one hand, the results for REU show that the wood-based process configurations use the feedstock more efficiently (Fig. 10a). This is mostly due to the assumed losses of feedstock during transportation from the forest (wood) or field (straw) to the production site (0 % in the case of wood, 15 % in the case of straw). It should be noted here that the transportation distances for both feedstocks were assumed to be the same (at 25 km). This is however generally not the case, but can be justified for this study since the focus is on the conversion of the feedstock into fuel. On the other hand, the wood-based configurations use more fossil-based resources (or more NREU). The REU and NREU allocated to the ethanol produced and the total REU and NREU for the two feedstocks show a difference in how the two feedstocks perform relative to each other (compare Figs. 8, 10). This difference can be explained by the fraction of the environmental burden that is allocated to the ethanol product in case of the two feedstocks.

## Conclusions

A LCA was performed for a high-gravity technology in development that produces ethanol from spruce wood chips. The LCA was based on lab experiments in which a total of 30 different process configurations using different detoxification strategies were tested. The results show that the configurations in which the pretreated wood slurry is washed have the lowest environmental impact. These configurations were run at 20 % DM, whereas the other experiments were run at 30 % DM. A lower dry matter content may thus improve the environmental impact, although an even lower DM content may lead to additional use of fossil fuel to meet the process’ energy needs, specifically in downstream processing in order to remove the water from the main product stream. The main contributor to the environmental impact is the production and use of enzyme in all tested process configurations. It was demonstrated that either enzyme recycling or on-site production of enzyme can significantly reduce the environmental impact of the technology in development.

For the current case, the assessed impact categories [global warming (GWP), eutrophication (EP), acidification (AP) and photo-chemical ozone creation (POCP) potentials] show a strong linear correlation with the ethanol yield of each process configuration: the higher the yield, the lower the environmental impact. Furthermore, these impact categories showed a very strong cross-correlation which suggests that using GWP would suffice in the current case to accurately assess the environmental impact of the technology in development.

Finally, a comparison was done with an LCA of a similar technology in development that uses wheat straw as the feedstock based on the renewable (REU) and NREU of the tested process configurations. While a difference between the use of spruce wood chips or wheat straw can be discerned when considering REU and NREU, there is no statistically significant difference in the performance of the high-gravity technology between the use of these two feedstocks when considering the resulting GWP, AP and POCP. This is due to the high contribution of enzyme production and use to each of these impact categories for both spruce wood chips (67–77 % for GWP, 64–75 % for EP, 43–64 % for AP, and 72–87 % for POCP) and wheat straw (72–85 % for GWP, 37–48 % for EP, 37–69 % for AP, and 91–97 % for POCP, see [15]). However, using wheat straw as the feedstock leads to a higher EP which is due to the use of fertilizer during wheat cultivation.

The LCA points out the environmental hotspots where further development efforts will result in a reduction of the environmental impact of the process. The main identified hotspot in this study was enzyme production and use, which may be reduced by recycling the enzyme or by on-site production of the enzyme. While the results show a strong correlation among the impact categories in the current case study, one should be cautious to exclude impact categories in the assessment. Knowledge of the system under study and the causal links that may exist between inputs to and outputs from the system, and the impact categories provide further input for choosing the most relevant impact categories. The results in this study show that comparing an agricultural feedstock with a forestry feedstock needs to include EP, next to GWP.

## Additional file

10.1186/s13068-016-0468-3 Life cycle inventory and life cycle impact assessment results.

## Abbreviations

AD: anaerobic digestion
AP: acidification potential
COD: chemical oxygen demand
DM: dry matter
EP: eutrophication potential
FPU: filter paper unit
GWP: global warming potential
LCA: life cycle assessment
LCIA: life cycle impact assessment
NREU: non-renewable energy use
PEI: polyethylenimine
POCP: photochemical ozone creation potential
PSSF: pre-saccharification and fermentation
REU: renewable energy use
SHF: separate hydrolysis and fermentation
SSF: simultaneous saccharification and fermentation
WIS: water-insoluble solids
