# Supplementary material for: Life cycle impacts of ethanol production from spruce wood chips under high-gravity conditions
Source: Biotechnol Biofuels. 2016 Mar 5;9:53. doi: 10.1186/s13068-016-0468-3 (PMC4779266; doi:10.1186/s13068-016-0468-3)
Supplement: Supplementary file 1 — 10.1186/s13068-016-0468-3 Life cycle inventory and life cycle impact assessment results. [file 13068_2016_468_MOESM1_ESM.pdf]

1 Inventory data: Mass flows

Table 1
Final ethanol yield and mass balance results for the production of 1 L (= 0.79 kg) of ethanol for all of the tested process configurations

Detoxification strategy	T [°C]	DM [%]	Process strategy	Yield [%]	Ethanol conc. [%(w/w)]	Wood input [kg]	Methane [kg]	Lignin, product [kg]	Non-digested cellulose [kg][1]
Base case	30	12	PSSF	73	2.8	7.4	0.03	0	0.80
Adaptation	30	30	PSSF	27	2.5	19.5	0.34	1.0	3.1
Adaptation	34	30	PSSF	10	0.9	54.6	1.4	4.5	8.7
Adaptation	37	30	PSSF	8	0.7	68.0	1.9	6.1	10.9
Adaptation	40	30	PSSF	4	0.4	137	4.0	14.2	21.8
Adaptation	30	30	SHF	37	3.4	14.4	0.35	1.0	1.4
Adaptation	40	30	SHF	2	0.2	273	11.3	36.3	26.5
Adapt + nutrients	30	30	PSSF	53	4.9	10.1	0.05	0.12	1.6
Adapt + nutrients	34	30	PSSF	41	3.8	13.0	0.14	0.44	2.1
Adapt + nutrients	37	30	PSSF	24	2.2	22.7	0.44	1.4	3.6
Adapt + nutrients	40	30	PSSF	12	1.1	45.5	1.1	3.4	7.2
Adapt + nutrients	30	30	SHF	61	5.6	8.8	0.11	0.27	0.85
Adapt + nutrients	40	30	SHF	10	0.9	54.6	2.1	6.2	5.3
$Na_2S_2O_4$ detox	30	30	PSSF	57	5.2	9.4	0.04	0.08	1.4
$Na_2S_2O_4$ detox	34	30	PSSF	57	5.2	9.4	0.04	0.08	1.4
$Na_2S_2O_4$ detox	37	30	PSSF	51	4.7	10.5	0.06	0.14	1.7
$Na_2S_2O_4$ detox	40	30	PSSF	47	4.3	11.4	0.09	0.24	1.8
$Na_2S_2O_4$ detox	30	30	SHF	59	5.4	9.1	0.12	0.31	0.88
$Na_2S_2O_4$ detox	40	30	SHF	41	3.8	13.0	0.29	0.86	1.3
Washing slurry	30	20	PSSF	59	3.8	12.9	0.27	0.79	1.4
Washing slurry	34	20	PSSF	61	3.9	12.6	0.27	0.77	1.4
Washing slurry	40	20	PSSF	65	4.1	11.9	0.25	0.72	1.2
Batch, slurry	35	30	SSF	3	0.2	192	5.5	19.2	32.1
Batch, solids	35	30	SSF	14	0.9	42.4	1.0	2.7	7.1
Fed-batch	35	30	SSF, all	59	4.0	10.1	0.08	0.18	1.5
Fed-batch	35	30	SSF, excl. yeast	41	2.8	14.4	0.16	0.46	2.4
Fed-batch	35	30	SSF, excl. enzyme	49	3.3	12.2	0.10	0.25	2.0
PEI detox	34	30	SSF	67	5.1	9.6	0.04	0.12	1.4
PEI detox	34	30	SHF, pre-hydro	46	3.5	13.9	0.28	0.81	1.5
PEI detox	34	30	SHF, post-hydro	50	3.8	13.0	0.26	0.75	1.3

[1] The non-digested cellulose was treated as a material loss (it is neither recycled nor incinerated).

2 Inventory data: Energy flows

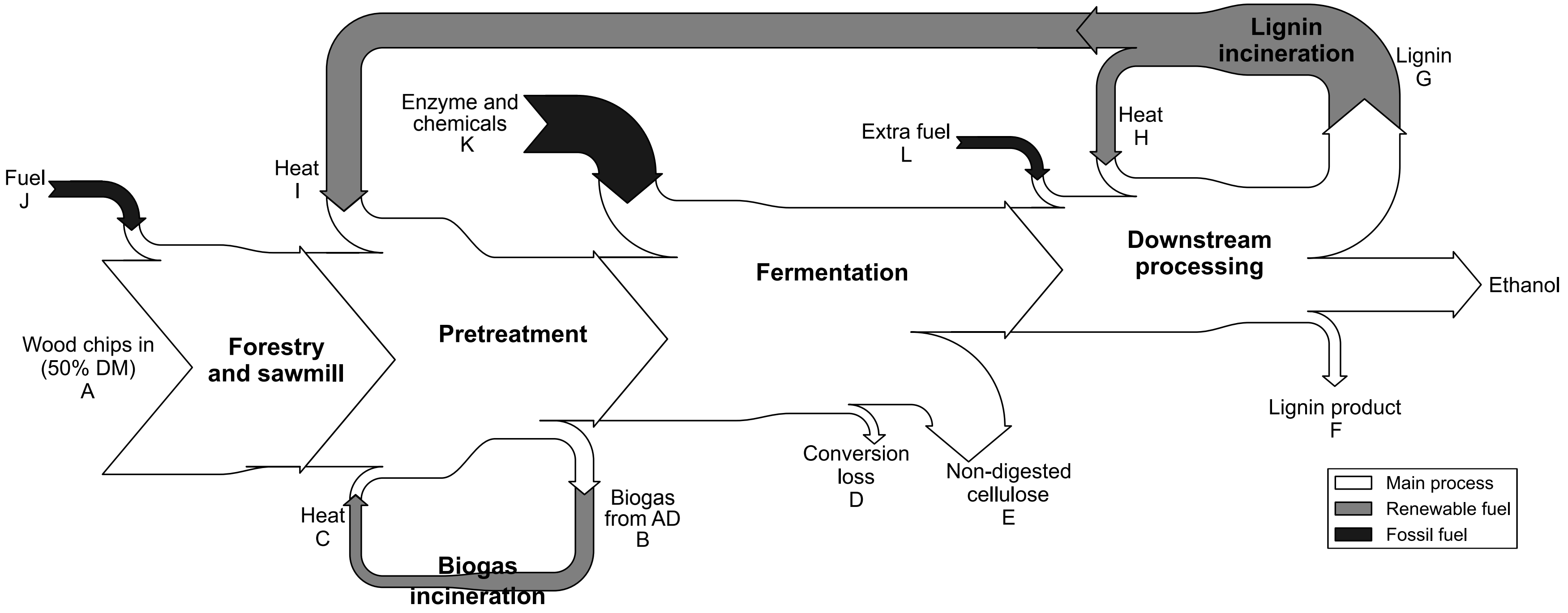

Fig. 1. Energy flows in the analyzed systems. The letters signify the flows as given in Table 2.

Table 2
Final ethanol yield and energy balance results for the production of 1 L (= 23.4 MJ) of ethanol for all of the tested process configurations.

Detoxification strategy	T [°C]	DM [%]	Process strategy	Yield [%]	Renewable resources A	B	C	D	E	F	G	H	I	Fossil resources J	K	L
Base case	30	12	PSSF	73	71	2	2	7	14	0	24	9	9	7	36	3
Adaptation	30	30	PSSF	27	187	19	17	28	53	25	37	16	12	15	93	0
Adaptation	34	30	PSSF	10	525	79	71	94	148	107	68	42	9	44	260	0
Adaptation	37	30	PSSF	8	653	103	92	119	184	146	71	46	7	56	322	0
Adaptation	40	30	PSSF	4	1315	221	199	248	370	340	97	72	2	112	649	0
Adaptation	30	30	SHF	37	138	19	17	24	40	24	21	12	4	12	68	0
Adaptation	40	30	SHF	2	2616	630	567	613	450	870	0	0	0	222	1295	0
Adapt + nutrients	30	30	PSSF	53	97	3	2	10	27	3	29	10	12	8	49	0
Adapt + nutrients	34	30	PSSF	41	125	8	7	16	35	11	31	11	12	10	63	0
Adapt + nutrients	37	30	PSSF	24	218	24	22	34	61	32	40	19	11	18	111	0

Continued on next page

Table 2 – *Continued from previous page*

Detoxification strategy	T [°C]	DM [%]	Process strategy	Yield [%]	Renewable resources A	B	C	D	E	F	G	H	I	Fossil resources J	K	L
Adapt + nutrients	40	30	PSSF	12	436	64	57	77	123	83	62	38	9	37	222	0
Adapt + nutrients	30	30	SHF	61	85	7	6	11	15	7	22	9	7	7	43	0
Adapt + nutrients	40	30	SHF	10	525	114	103	116	90	149	26	19	0	45	369	0
$Na_2S_2O_4$ detox	30	30	PSSF	57	90	2	2	10	24	2	28	10	12	7	46	0
$Na_2S_2O_4$ detox	34	30	PSSF	57	90	2	2	10	24	2	28	10	12	7	46	0
$Na_2S_2O_4$ detox	37	30	PSSF	51	101	3	3	11	28	3	30	10	12	8	51	0
$Na_2S_2O_4$ detox	40	30	PSSF	47	109	5	4	13	31	6	30	10	12	9	55	0
$Na_2S_2O_4$ detox	30	30	SHF	59	87	7	6	12	15	8	21	9	7	7	45	0
$Na_2S_2O_4$ detox	40	30	SHF	41	125	16	14	21	21	21	21	11	5	10	64	0
Washing slurry	30	20	PSSF	59	124	18	14	10	24	19	22	12	5	10	46	0
Washing slurry	34	20	PSSF	61	121	18	14	10	23	18	22	11	5	45	10	0
Washing slurry	40	20	PSSF	65	114	19	14	9	20	17	21	11	5	42	9	0
Batch, slurry	35	30	SSF	3	1846	304	273	375	545	460	154	108	8	158	756	0
Batch, solids	35	30	SSF	14	407	56	50	76	120	65	70	41	12	34	167	0
Fed-batch	35	30	SSF, all	59	97	5	4	13	25	4	28	10	11	8	39	0
Fed-batch	35	30	SSF, excl. yeast	41	139	9	8	20	41	11	35	13	13	11	57	0
Fed-batch	35	30	SSF, excl. enzyme	49	117	5	5	16	35	6	33	12	13	9	48	0
PEI detox	34	30	SSF	67	92	3	2	11	24	3	28	9	12	7	46	0
PEI detox	34	30	SHF, pre-hydro	46	133	16	14	23	26	20	25	12	6	11	66	0
PEI detox	34	30	SHF, post-hydro	50	125	14	13	21	23	18	23	12	6	10	62	0

3 Life cycle impact assessment and process water removal

Table 3
Final ethanol yield, ethanol concentration, life cycle impact assessment results and water removal during downstream processing for the production of 1 L (= 0.79 kg) of ethanol for all of the tested process configurations.
GWP = Global Warming Potential; EP = Eutrophication Potential; AP = Acidification Potential; POCP = Photochemical Ozone Creation Potential.

Detoxification strategy	T [°C]	DM [%]	Process strategy	Yield [%]	Ethanol conc. [%(w/w)]	GWP [kg CO_2-eq]	EP [kg NO_x-eq]	AP [kg SO_2-eq]	POCP [kg C_2H_4-eq]	Water removal [L water]
Base case	30	12	PSSF	73	2.8	3.4	6.0×10^{-3}	3.3×10^{-2}	2.3×10^{-3}	25
Adaptation	30	30	PSSF	27	2.5	4.9	8.5×10^{-3}	3.7×10^{-2}	3.1×10^{-3}	22
Adaptation	34	30	PSSF	10	0.9	6.0	1.0×10^{-2}	4.5×10^{-2}	3.8×10^{-3}	62
Adaptation	37	30	PSSF	8	0.7	5.9	9.9×10^{-3}	4.3×10^{-2}	3.7×10^{-3}	76
Adaptation	40	30	PSSF	4	0.4	5.8	9.7×10^{-3}	4.2×10^{-2}	3.7×10^{-3}	156
Adaptation	30	30	SHF	37	3.4	3.7	6.2×10^{-3}	2.7×10^{-2}	2.3×10^{-3}	16
Adaptation	40	30	SHF	2	0.2	4.4	7.3×10^{-3}	3.2×10^{-2}	2.8×10^{-3}	310
Adapt + nutrients	30	30	PSSF	53	4.9	3.9	7.0×10^{-3}	3.1×10^{-2}	2.5×10^{-3}	11
Adapt + nutrients	34	30	PSSF	41	3.8	4.2	7.4×10^{-3}	3.3×10^{-2}	2.7×10^{-3}	15
Adapt + nutrients	37	30	PSSF	24	2.2	5.0	8.4×10^{-3}	3.7×10^{-2}	3.1×10^{-3}	26
Adapt + nutrients	40	30	PSSF	12	1.1	6.0	1.0×10^{-2}	4.5×10^{-2}	3.8×10^{-3}	52
Adapt + nutrients	30	30	SHF	61	5.6	3.1	5.5×10^{-3}	2.4×10^{-2}	2.0×10^{-3}	10
Adapt + nutrients	40	30	SHF	10	0.9	4.7	8.0×10^{-3}	3.4×10^{-2}	3.0×10^{-3}	62
$Na_2S_2O_4$ detox	30	30	PSSF	57	5.2	3.8	7.0×10^{-3}	3.3×10^{-2}	2.5×10^{-3}	11
$Na_2S_2O_4$ detox	34	30	PSSF	57	5.2	3.8	7.0×10^{-3}	3.3×10^{-2}	2.5×10^{-3}	11
$Na_2S_2O_4$ detox	37	30	PSSF	51	4.7	4.1	7.5×10^{-3}	3.5×10^{-2}	2.7×10^{-3}	12
$Na_2S_2O_4$ detox	40	30	PSSF	47	4.3	4.1	7.6×10^{-3}	3.6×10^{-2}	2.8×10^{-3}	13
$Na_2S_2O_4$ detox	30	30	SHF	59	5.4	3.2	5.8×10^{-3}	2.8×10^{-2}	2.1×10^{-3}	10
$Na_2S_2O_4$ detox	40	30	SHF	41	3.8	3.6	6.3×10^{-3}	3.0×10^{-2}	2.4×10^{-3}	15
Washing slurry	30	20	PSSF	59	3.8	2.8	4.9×10^{-3}	2.3×10^{-2}	1.8×10^{-3}	16
Washing slurry	34	20	PSSF	61	3.9	2.8	4.8×10^{-3}	2.3×10^{-2}	1.7×10^{-3}	16
Washing slurry	40	20	PSSF	65	4.1	2.7	4.6×10^{-3}	2.2×10^{-2}	1.7×10^{-3}	15
Batch, slurry	35	30	SSF	3	0.2	5.3	8.8×10^{-3}	4.0×10^{-2}	3.3×10^{-3}	292
Batch, solids	35	30	SSF	14	0.9	5.5	9.7×10^{-3}	4.4×10^{-2}	3.5×10^{-3}	64
Fed-batch	35	30	SSF, all	59	4.0	3.2	5.8×10^{-3}	2.7×10^{-2}	2.0×10^{-3}	15
Fed-batch	35	30	SSF, excl. yeast	41	2.8	4.0	7.1×10^{-3}	3.1×10^{-2}	2.5×10^{-3}	22
Fed-batch	35	30	SSF, excl. enzyme	49	3.3	3.7	6.7×10^{-3}	3.1×10^{-2}	2.4×10^{-3}	18
PEI detox	34	30	SSF	67	5.1	3.4	6.5×10^{-3}	2.9×10^{-2}	2.0×10^{-3}	11
PEI detox	34	30	SHF, pre-hydro	46	3.5	3.6	6.6×10^{-3}	2.9×10^{-2}	2.1×10^{-3}	16
PEI detox	34	30	SHF, post-hydro	50	3.8	3.4	6.3×10^{-3}	2.8×10^{-2}	2.0×10^{-3}	15

4 Correlation between fermentation temperature and environmental impact

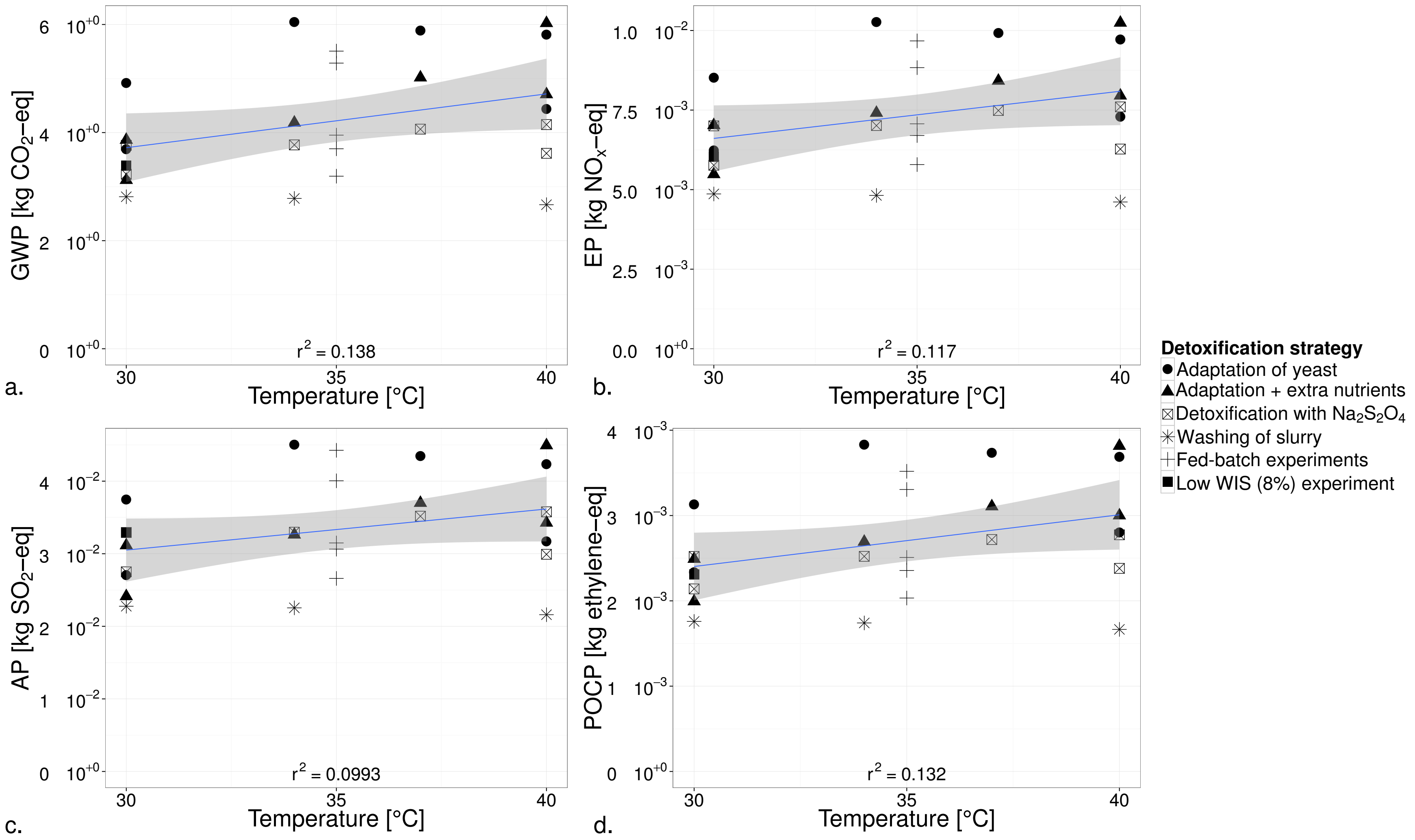

Fig. 2. Correlation between the fermentation temperature of the process configurations and their environmental impacts. The impact categories are: a. global warming potential (GWP); b. eutrophication potential (EP); c. acidification potential (AP); d. photochemical ozone creation potential (POCP). The linear trend line (in blue) and the 95 % confidence intervals (in grey) are given. The correlation coefficients (r^2) are mentioned in each graph.
